# Functional TRPA1 Channels Regulate CD56^dim^CD16^+^ NK Cell Cytotoxicity against Tumor Cells

**DOI:** 10.3390/ijms241914736

**Published:** 2023-09-29

**Authors:** Fernanda Scopelliti, Valentina Dimartino, Caterina Cattani, Andrea Cavani

**Affiliations:** 1 National Institute for Health, Migration and Poverty INMP/NIHMP, Via di S.Gallicano, 25, 00153 Rome, Italycaterina.cattani@inmp.it (C.C.); andrea.cavani@inmp.it (C.A.); 2 National Institute for Infectious Diseases Lazzaro Spallanzani IRCCS, Via Portuense 292, 00149 Rome, Italy

**Keywords:** TRPA1, NK, immune system, calcium, cytotoxicity

## Abstract

Transient receptor potential ankyrin 1 (TRPA1) channels are expressed on the surface of different cell types, including immune cells. However, TRPA1’s role in the context of innate and adaptive immune responses has not been fully elucidated so far. In this study, we aimed at investigating the expression and function of TRPA1 channels on NK cells. Among NK cells, TRPA1 was highly expressed by the CD56^dim^CD16^+^ subpopulation, but not by CD56^bright^CD16^−^ cells, as detected by FACS. TRPA1 activation with the potent ligand allyl isothiocyanate (AITC) induces intracellular calcium flux in CD56^dim^CD16^+^ cells, which was prevented by the TRPA1 antagonist HC-030031. AITC treatment increased the membrane around NKp44 and strongly decreased CD16 and CD8 expression, while CD158a, CD159a, NKG2d, NKp46 were substantially unaffected. Importantly, AITC increased the granzyme production and CD107 expression and increased NK cell-mediated cytotoxicity towards the K562 cell line and two different melanoma cell lines. In parallel, TRPA1 activation also plays regulatory roles by affecting the survival of NK cells to limit uncontrolled and prolonged NK cell-mediated cytotoxicity. Our results indicate that the activation of TRPA1 is an important regulatory signal for NK cells, and agonists of TRPA1 could be used to strengthen the tumor response of the immune system.

## 1. Introduction

TRP channels are non-selective cation channels grouped in six subfamilies: canonical TRPs (TRPC), vanilloid TRPs (TRPV), melastatin TRPs (TRPM), mucolipin TRPs (TRPML), polycystin TRPs (TRPP) and ankyrin TRP (TRPA) [1,2,3,4]. TRPA1 is the only member of the TRPA family [5]; it can be activated by heterogeneous stimuli, including noxious cold temperature [6], the stretch of cell membrane [7], reactive irritants and various chemical compounds, such as mustard oil, cinnamaldehyde, ginger (gingerol), oregano (carvacrol), garlic (allicin), clove oil (eugenol), wintergreen oil (methyl salicylate) and allyl isothiocyanate (AITC), which is a spicy component of wasabi and the best recognized agonist of TRPA1 [8,9,10,11,12]. TRPA1 is also activated by endogenous molecules produced during oxidative stress, including hydroxyl radicals, 4-hydroxynonenal, cyclopentenone prostaglandins and hypochlorite [13]. All these molecules bind covalently cysteine residues of TRPA1, causing conformational change that opens the channel and induces depolarization of the cell, allowing Ca^++^ influx. Additionally, a number of nonelectrophilic compounds, such as menthol [14], nicotine [15] and delta9-tetrahydrocannabinol [9,16], act as agonists without interacting covalently with TRPA1 [17].

TRPA1 was first identified in nerve endings, but subsequently it has been detected in various cell types. Several studies indeed showed the presence of TRPA1 channels both in neuronal cells and non-neuronal tissues, such as vascular endothelial cells and smooth muscle cells, as well as immune system cells, including mast cells [18], human monocyte-derived immature dendritic cells (DCs) [19] and monocytes [6,7,9,20,21,22,23]. Given the wide distribution of the channel, TRPA1 can influence numerous regulatory and proinflammatory pathways [24,25].

Natural killer (NK) cells belong to group 1 innate lymphoid cells, representing between 5% and 15% of the total population of circulating lymphocytes [26]. The expression on the surface of CD56 (neural cell-adhesion molecule) and CD16 (FcγIII receptor), the low affinity receptor of IgG receptors, distinguished two NK subsets: CD56^bright^CD16^−^ and CD56^dim^CD16^+^ NK cells. They represent, respectively, 10% and 90% of peripheral blood NK cells. NK cells have many biological functions, ranging from immunomodulation capacities and direct cytotoxicity of virally infected and transformed cells. Different subtypes of NK cells play different roles in the immune system: in particular, CD56^bright^CD16^–^ NK cells act predominately as immunosurveillance cells with potent cytokine production, whereas CD56^dim^CD16^+^ NK cells are the fully mature counterpart with high cytotoxic potential.

Intracellular calcium (Ca^2+^) mobilization plays an important role in regulating different intracellular signaling pathways in NK cells, such as the antibody-dependent cellular cytotoxicity (ADCC) or mitogen-activated protein kinase pathway. This is necessary for the development of immune synapse, cytokine production and cytotoxic activity. Also, natural cytotoxicity of NK cells is mediated by granule polarization and degranulation whose mechanism requires intracellular (Ca^2+^) [27]. Therefore, identifying mechanisms that control the entry of calcium into NK cells may be important to enhance NK cell activity towards tumor cells and to prevent immunoevasion, as it is one of the major obstacles in anticancer therapy. Indeed, in recent years, several studies have been published claiming that impaired Ca^2+^ signaling leads to severely defective cytotoxic granule exocytosis accompanied by weak target cell lysis [28,29]. The aim of this study was to evaluate whether TRPA1 was expressed by NK cells and investigate the role of the channel in NK cell functions. Results show that TRPA1 channels are expressed in a subpopulation of CD56^dim^CD16^+^ NK and that the treatment with the specific ligand AITC increases NK activation and cytotoxicity.

## 2. Results

### 2.1. TRPA1 Is Expressed on the Surface of CD56^dim^CD16^+^ Natural Killer Cells

**Figure 1:**
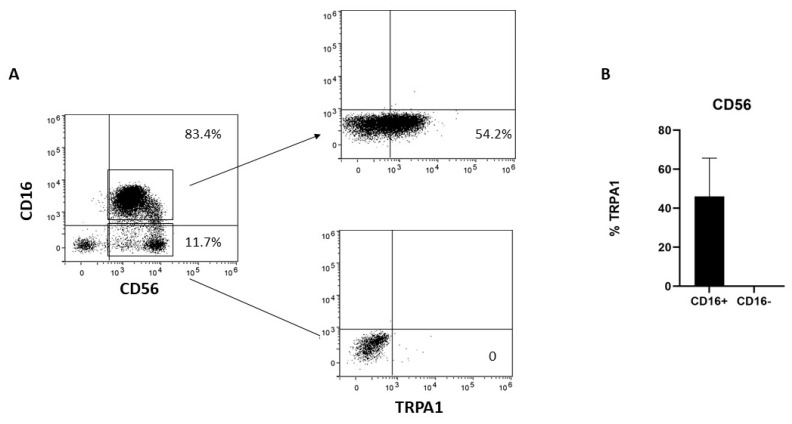
TRPA1 expression in NK cells. (**A**) NK cells obtained from six different donors were collected, stained with the mAbs, and the expression of surface markers analyzed by FACS, as described in Section 4. The plots show the data obtained in one representative experiment out of six. (**B**) Graphs show the percentage of TRPA1+ in CD56^dim^CD16^+^ and CD56^bright^CD16^−^ NK cells in six different donors.

Human NK cells can be subdivided into two major subsets; namely, the CD56^dim^CD16^+^ cytotoxic NK cells and the CD56^bright^CD16^−^ NK cells. To better characterize TRPA1 distribution in NK cells, the cells were negatively selected with NK cell magnetic beads and then analyzed by cytofluorimetry. TRPA1 was expressed only in the CD56^dim^CD16^+^ fraction, whereas CD56^bright^/CD16^−^ lack membrane-associated TRPA1 (Figure 1).

**Figure 2:**
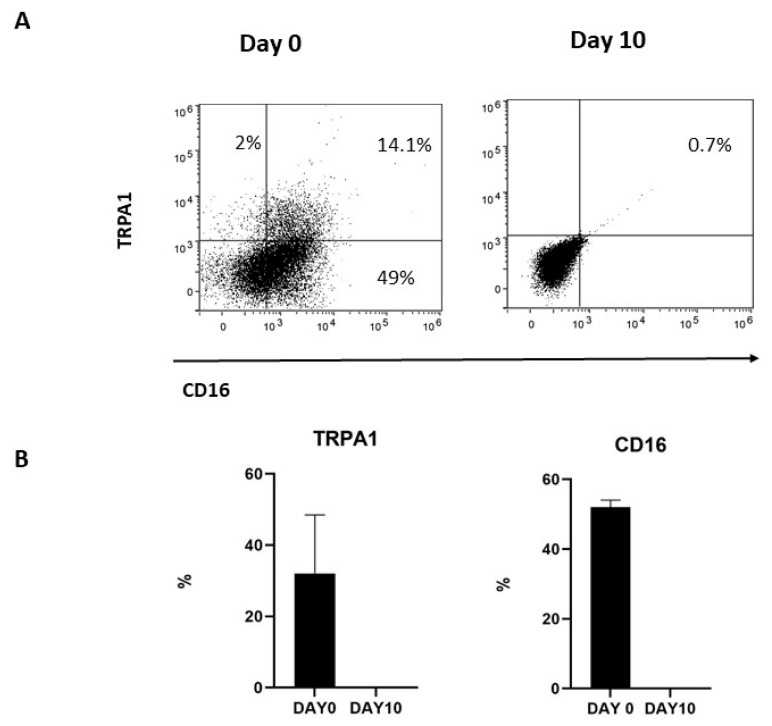
(**A**) TRPA1 expression was positive correlated with CD16 expression in cultured NK cells. NK cells obtained from three donors were cultured at selected times, and (**B**) the expressions of TRPA1 and the CD16 receptor were analyzed by FACS, as described in Methods. *Graphs show the percentage of TRPA1+ and CD16+ NK cells at day 0 and day 10 in three different donors*.

To investigate whether membrane-associated TRPA1 was correlated with the expression of CD16, TRPA1^+^CD56^+^CD16^+^ were repeatedly stimulated with IL-2 and, after 10 days, the expression of the two markers was investigated by FACS. As previously reported [30], in vitro activated NK cells progressively decrease CD16 expression. Meanwhile, membrane-associated TRPA1 was lost during culturing, thus indicating a correlation between CD16 and TRPA1 expression for NK cells (Figure 2).

### 2.2. Membrane-Associated TRPA1 on CD56^dim^CD16^+^ NK Cells Is Functionally Active

**Figure 3:**
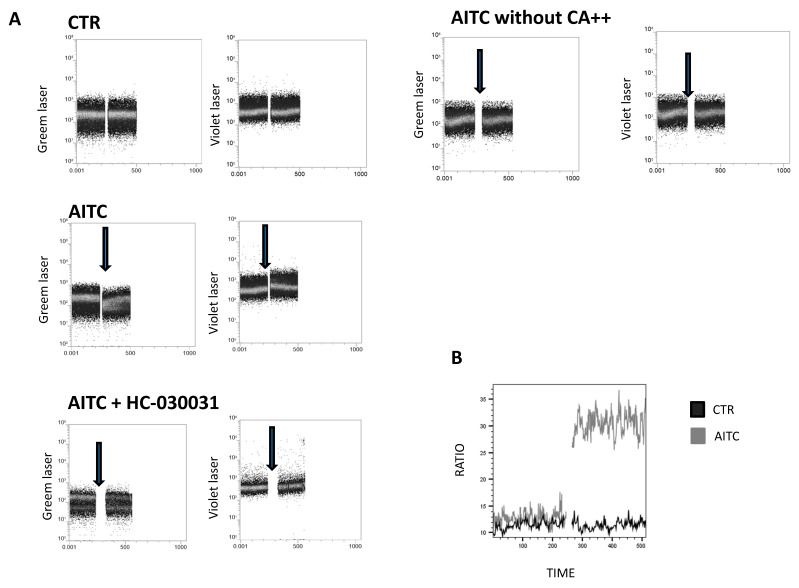
Calcium assay to monitor the calcium influx in NK-treated cells. (**A**) NK cells were loaded with Fura red AM for 30 min. Intracellular calcium mobilization in response to AITC and HC-030031(100 μM) was measured by flow cytometry. Following the treatment of NK cells with AITC, HC-030031 or 0.01% DMSO, used as the control, calcium entry into the cell cytoplasm was detected by an increase in the fluorescence signal from the violet laser, whereas fluorescence detected by an increase in the signal from the green laser shows the decrease in quenched cytoplasmic calcium. The single cell suspension was monitored for 240 s (background signal), prior to the addition of AITC or HC-030031, to establish non-specific fluctuations in the intracellular calcium. Arrows indicate the addition of substances. (**B**) Calcium flux is depicted as the mean value of the Fura red ratio over time in response to AITC.

To examine whether TRPA1 channels were functional, calcium flux was measured by Fura red in freshly isolated, NK cells upon stimulation with the TRPA1 ligand allyl isothiocyanate (AITC). In the presence of extracellular Ca^++^, AITC 100 μM induced a rapid increase in intracellular Ca^++^ (Figure 3A). In contrast, AITC did not affect Ca^++^ in the absence of extracellular Ca^++^ (Figure 3A). Pretreatment of NK cells with the TRPA1 antagonist HC-030031 (100 μM) completely prevented the intracellular Ca^++^ flux induced by AITC (Figure 3A), thus indicating that the effect of AITC is correlated with TRPA1 binding.

Our data show that AITC induces a substantial increase in calcium into the cell cytoplasm compared to the control (Figure 3B).

### 2.3. TRPA1 Activation Modulates NK Receptors

NK cells express several inhibitory and activating receptors that can regulate their function upon interaction with specific ligands on the surface of transformed, virus-infected, or stressed cells. To evaluate whether triggering TRPA1 on NK cells could modulate their receptor repertoire, dose-response tests (1-5-10 μM, data not shown) were performed with the aim of defining the more efficient AITC concentration to add to NK cultures. For this purpose, 10 μM concentration was chosen and used in the following experiments.

NK cells were cultured for 24 h alone or with AITC and the expression of surface receptors was analyzed by cytofluorimetry.

**Figure 4:**
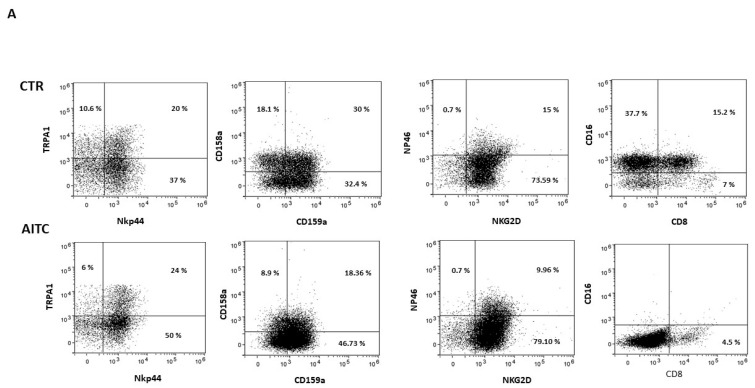

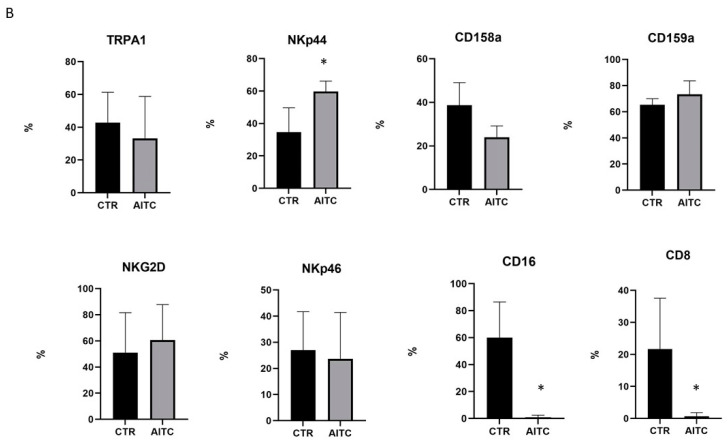
AITC-induced modulation of the NK receptors. (**A**) NK obtained from three different donors were cultured 24 h in the presence or in the absence of AITC and analyzed by FACS, as described in Methods. (**B**) Graphs show the percentage of CD107 + cells treated with AITC or untreated (* *p* < 0.05) in three different donors.

As shown in Figure 4, 24 h treatment with 10 μM AITC increased the expression of the NKp44 marker and non-significantly decreased CD158a, whereas CD159a, NKG2d, NKp46 were substantially unaffected (Figure 4). Surprisingly, AITC induced a strong decrease in both CD16 and CD8 receptors (Figure 4), which was expressed, respectively, by 52.9 and 22.2% of the untreated CD56^dim^CD16^+^.

### 2.4. TRPA1 Activation Induced NK Degranulation and Tumor Cell Apoptosis

**Figure 5:**
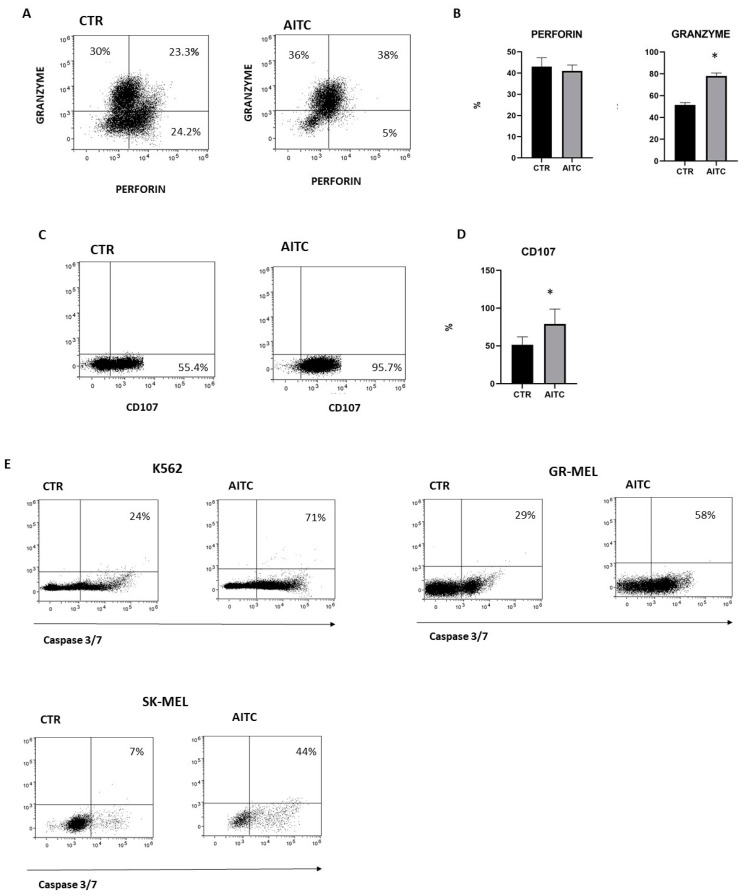
TRPA1 activation induced degranulation of NK cell and tumor apoptosis. (**A**) NK cells from three donors were treated or not with AITC and cocultured with K562 cells and monitored for perforin and granzyme production by flow cytometry, as described in Methods. (**B**) Graphs show the percentage of granzyme and perforin in NK cells treated with AITC or untreated in three different donors (* *p* < 0.05). (**C**) NK cells from three donors were treated or not with AITC and cocultured with K562 cells and the expression of CD107 was evaluated by flow cytometry, as described in Methods. (**D**) Graphs show the percentage of CD107 + cells treated with AITC or untreated from each donor (* *p* < 0.05). (**E**) NK cells were exposed to AITC 10 μM, or left untreated, and cocultured 6 h with K562, GR-MEL, SKMEL. The induction of apoptosis was determined by FACS as the percentage of caspase-3 and -7-positive tumor cells. One experiment of three performed is shown.

NK cytotoxicity of cellular targets is mostly mediated by the release of lytic granules that contain granzymes and perforin. The release of lytic granules by NK cells is paralleled by the CD107a expression, which is considered a reliable marker for the cytotoxic capability of NK cells. To investigate whether TRPA1 triggering on NK cells could affect their cytotoxic capacity, purified NK cells, pretreated or not with AITC 10 μM, were washed extensively and subsequently were cocultured with K562 6 h at 37° and perforin, while granzyme and CD107 expression were investigated by FACS [31]. The results demonstrated an increase in the number of cells which produce granzyme as compared to untreated NK cells (Figure 5A). Moreover, NK cells treated with AITC expressed higher levels of CD107 (Figure 5B,C).

In light of this observation, we studied the capacity of NK cells to kill tumor cells lines in vitro cytotoxicity assays since a direct role is strongly suggested of NK cells in the induction of tumor apoptosis. As expected, NK-induced apoptosis of K562 and two melanoma cell lines was increased by AITC treatment, as determined by the quantification of the apoptosis markers caspase-3 and -7 (Figure 5D).

Instead, AITC does not affect the NK cell production of IFN-γ and TNF-α (data not shown).

**Figure 6:**
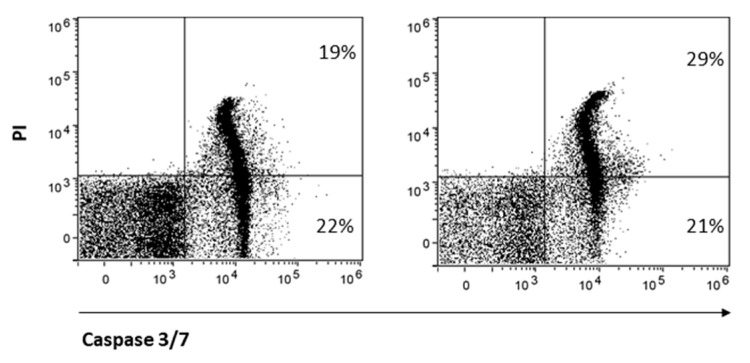
AITC-induced apoptosis of NK.

Finally, CD8 is reported to protect NK cells from apoptosis [32]. Therefore, we hypothesized that treatment with AITC, responsible for strong reduction in CD8 expression, may also influence NK cell survival. AITC exposure only partially reduced NK cell survival, as determined by the quantification of the markers caspase-3 and -7 and PI (Figure 6).

## 3. Discussion

The results of the present study demonstrate that CD56^dim^CD16^+^ NK cells display functional membrane-bound TRPA1 ion channels, as indicated by Ca^2+^ influx assays when triggered by the specific ligand AITC. TRPA1-mediated NK activation determines the upregulation of CD107 and NKp44 and also increases granzyme expression and the capacity of NK cells to kill both K562 cells and melanoma cell lines.

Most of the investigation of TRPA1 has been conducted in sensory neurons. In those cells, TRPA1, together with TRPV1, exerts excitatory function mediating itching, pain and the release of pro-inflammatory neuropeptides, thus modulating neurogenic inflammatory processes. Evidence exists that TRPA1 is also expressed not only in neuronal cells but also in immune system cells, such as mast cells, human monocytes, T cells from the GI tract, and Langerhans cells. However, the role of TRP in immune cells has not yet been fully elucidated. Additionally, complexity deriving from the multitude of agonistic ligands, which can activate the channel through both covalent and non-covalent binding, limits functional studies of TRPA1 in various cell types.

Studies in human lung fibroblasts have showed that TRPA1 expression is upregulated by TNF-α and that TRPA1 ligands upregulate the release of IL-8, thus contributing to lung inflammation [33].

In contrast, the IL-1β expression in macrophages was suppressed by the TRPA1 agonist cinnamaldehyde [34] and the TRPA1 agonist cannabinoid reduces INF-γ in macrophages by inhibiting nitric oxide (NO), thus exerting an anti-inflammatory role in colitis in mice [35].

Among CD56^+^NK cells, we found that TRPA1 is exclusively expressed by the CD56^dim^CD16^+^ subset, which represents mature NK cells with high cytotoxic potential. Interestingly, the expression of TRPA1 and CD16 appears strictly correlated, since progressive loss of CD16 by NK cells activated during the culture procedures also decreases TRPA1 surface expression. Several studies demonstrated that cytokine exposure or NK cell activation followed contact with tumor target cells which led to marked decreases in CD16 expression, and this effect is at least in part mediated by metalloproteinase [30].

TRP channels are involved in intracellular Ca^2+^ influx, thus regulating a multitude of Ca-dependent cellular functions, including cell proliferation and differentiation and the release of cytokines and chemokines. Moreover, intracellular Ca^2+^ influx activates cytoskeleton movement that facilitates the secretory vesicles to fuse with the plasma membrane to ultimately form the immune synapse [1,36,37].

Activation of TRPA1 by AITC, a potent and selective TRPA1 ligand, showed a significant augmentation of intracellular Ca^2+^, which was prevented by the pretreatment with the antagonist of the calcium channel HC-030031, as reported in cellular models of other studies [9,38]. Moreover, treatment of activated NK cells with AITC increased the expression of NPK44, whereas CD158a was decreased and NKG2D, CD159a and NKP46 were substantially unaffected. Although many structurally distinct receptors are involved in NK-cell effector functions activation, it is not yet clear if a single receptor is necessary or sufficient to activate NK cells nor to what extent the expression of other receptors may be redundant. Specifically, NKp44 is a natural cytotoxic receptor involved in the recognition of multiple ligands on the surface of tumor cells and virus-infected cells.

Granule exocytosis-mediated target cell killing is a Ca^2+^-dependent process. Consistently, we hypothesize that TRPA1, regulating the NK Ca^2+^ influx, could affect NK cytotoxicity. The accumulation of activating signals that overwhelms the inhibitory signals triggers NK cell-mediated cytotoxicity [39]. With the initiation of NK cell cytotoxicity, NK cells secrete perforin and granzyme, thus forming membrane pores on the target membrane and causing apoptosis through caspase activation [40]. In our experiments, the stimulation of TRPA1 turns NK cells into efficient killers, which require the rapid synthesis, safe trafficking and storage of large amounts of cells that produces perforin and granzyme. Supporting these data, NK cells treated with AITC are able to induce apoptosis in different types of tumor cells lines.

AITC treatment reduces cell viability but only to a small extent of the activated NK cells. The loss of CD8 that follows TRPA1 activation may be involved in the increased NK cell susceptibility to apoptosis, since, as previously reported, the CD8 molecule provides NK cells with a survival mechanism after target cell lysis [32]. Although this mechanism requires further investigation, it appears that repeated treatment could be a tool to limit prolonged and uncontrolled NK cell-mediated cytotoxicity that could lead to excessive damage in the surrounding tissue due to the release of pro-inflammatory substances, such as ATP, by apoptotic tumor cells.

Taken together, these data indicate that TRPA1 channels are essential components of calcium signaling in NK cells, and that TRPA1 agonists may represent a novel strategy to enhance NK cell functions against tumors.

NK cells were treated or not with AITC 10 μM for 24 h. The induction of apoptosis was determined by FACS as a percentage of caspase-3 and -7 cells and PI.

## 4. Materials and Methods

### 4.1. Cell Cultures

Peripheral blood mononuclear cells (PBMC) were isolated through Ficoll-Hypaque (Cedarlane, Burlington, ON, Canada) after gradient centrifugation starting from buffy coats collected from the Transfusional Unit of the S.Camillo Forlanini hospital (Italy, Rome). NK cells were obtained by negative selection with NK cell magnetic beads according to the manufacturer’s protocol (Miltenyi, Bergisch Gladbach, Germany). The resulting NK cells (>90% CD56^+^) were suspended in complete RPMI supplemented with 10% FBS (HyClone, Logan, UT, USA) and maintained in culture with rhIL-2 (100 U/mL) at 37 °C, 5% CO_2_. To evaluate the effect of AITC on receptor expression, NK cells were activated with rhIL-2 (100 U/mL) and cultured with or without AITC 10 μM for 24 h.

### 4.2. Fura Red Analysis

NK cells were resuspended at 1 × 10^7^ cells/mL in 37 °C PBS with 1 μM of Fura red AM (Invitrogen, Altrincham, UK) and incubated at 37 °C for 30 min. Cells were washed and resuspended to 1 × 10^7^ cells/mL in RPMI with 1% FBS; cells were allowed to equilibrate for 10 min at 37 °C and subsequently analyzed by flow cytometry. Background, non-specific calcium flux was recorded for 240 s; then the cells were treated with Allyl isothiocyanate (AITC, Sigma Aldrich, MO, USA) 100 μM in the presence or absence of HC-030031 (Sigma Aldrich, St. Louis, MO, USA) 100 μM. The continuous recording rate was 8000–10,000 events/second for 240 s in total. Ratiometric analysis of Fura red is calculated by the ratio between the violet laser (406 nm) and the green laser (532 nm). The ratiometric ‘Fura red ratio’ was calculated as the increasing signal stimulated by the violet laser over the decreasing signal stimulated by the green laser (406 nm/532 nm), using the Kinetics tool in Flow Jo software version 10.8.1 (Tree Star Inc., Ashland, OR, USA).

### 4.3. Flow Cytometry Analysis

For surface marker staining, NK cells were washed with PBS and stained with FITC, PE- and PerCP-conjugated mAb for 20 min; the mAb anti-human CD56 (NCAM16.2), CD16 (B73.1), CD158a (HP-3E4), Nkp44 (p44-8), CD8 (SK1) (all from BD Biosciences, San Jose, CA, USA), CD159a (131,411 purchased from R&D Systems, Minneapolis, MN, USA), NKG2D (1D11) and NKp46 (9E2) (all purchased from Biolegend, San Diego, CA, USA) and TRPA1 (Alomone, Israel) were used followed by anti-rabbit PE (R&D) as a secondary antibody. Mouse IgG isotypes were used as controls (BD Biosciences). For acquisition and analysis, the first was performed using an Attune Nxt (Life Technologies, Carlsbad, CA, USA) cytofluorimeter whereas the second was performed using Flow logic software 7.1 (Miltenyi), according to guidelines for the use of flow cytometry and cell sorting in immunological studies [41].

### 4.4. Degranulation Assay

NK cells were incubated with K562 cells (ATTC) for 6 h at 37 °C. After 2 h incubation, brefeldin (1 μg/mL, BD Bioscience) was added for the last 4 h. Cells were collected and stained with surface antibodies for 20 min followed by Cytofix/Cytoperm (BD Biosciences) treatment and finally stained for 20 min with the indicated intracellular antibodies mAb in the presence of Perm/Wash solution (BD Biosciences). For acquisition, Attune Nxt (Life Technologies) cytofluorimetry was used whereas, for analysis, Flow logic software 7.1(Miltenyi) was used. CD107 (H4A3) and Granzyme (GB11) were purchased from BD Biosciences, Perforin (PRF1) was purchased from Biolegend, and mouse IgG isotype controls were purchased from BD Biosciences.

### 4.5. Cytotoxicity Assay

NK cells were pretreated or not with AITC 10 μM for 1 h and then cocultured with lymphoblastic K562 cell lines (ATTC) or two melanoma cell lines: GRMel and SKMel (kindly gifted by Cristina Failla, IDI IRCCS, Italy, Rome). The tumor cells were used as target cells for NK cell-mediated cytotoxicity. Caspase-3 and -7, of the fluorochrome-labeled inhibitors of caspases assay kit (FLICA; ImmunoChemistry Technologies, Bloomington, MN, USA), determined target cell apoptosis by FACS [42].

### 4.6. NK Apoptosis

NK cells were suspended in RPMI supplemented with 10% fetal calf serum and rhIL-2 (100 U/mL) and treated or not with AITC 10 μM for 24 h. Caspase-3 and -7, of the fluorochrome-labeled inhibitors of caspases assay kit with propidium (FLICA), determined NK cell apoptosis by FACS.

## Data Availability

Not applicable.

## Abbreviations

TRPA1: transient receptor potential ankyrin 1; NK: natural killers; AITC: allyl isothiocyanate; TRPV: vanilloid TRPs; TRPM: melastatin TRPs; TRPML: mucolipin TRPs; TRPP: polycystin TRPs; Ca^2+^: calcium; PBMC: peripheral blood mononuclear cells.
